# Neoadjuvant Therapy to Enable Partial Nephrectomy in Patients With High-Complexity or Locally Advanced Non-metastatic Renal Cell Carcinoma: A Systematic Review and Meta-Analysis

**DOI:** 10.7759/cureus.97247

**Published:** 2025-11-19

**Authors:** Fadi Abusal, Abdullah Alawadi, Mazen Alsharief, Ahmed Zeid, Saja Obeidat, Mohammad Wreikat, Hosam Serag

**Affiliations:** 1 Urology, Queen Elizabeth Hospital Birmingham, Birmingham, GBR; 2 Urology, International Postgraduate Medical Training Scheme, University Hospitals Birmingham, Birmingham, GBR; 3 Colorectal Surgery, Queen Elizabeth Hospital Birmingham, Birmingham, GBR; 4 Pediatrics, Yarmouk University of Jordan, Irbid, JOR; 5 Internal Medicine, Jordanian Royal Medical Services, Amman, JOR

**Keywords:** immune-checkpoint inhibitors, neoadjuvant therapy, nephron-sparing surgery, partial nephrectomy, renal cell carcinoma, tumor downstaging, tyrosine kinase inhibitors

## Abstract

This systematic review and meta-analysis evaluated the role of neoadjuvant systemic therapy in enabling partial nephrectomy (PN) for patients with high-complexity or locally advanced non-metastatic renal cell carcinoma (RCC). Thirteen studies, including 359 patients, were analysed, with most investigating vascular endothelial growth factor receptor-targeted tyrosine kinase inhibitors (VEGFR-TKIs), while some assessed immune checkpoint inhibitors (IO) alone or in combination. The pooled results showed that neoadjuvant therapy increased the feasibility of PN, achieving high rates of tumour shrinkage and negative surgical margins. Perioperative outcomes, including blood loss and complications, were comparable to upfront surgery, and severe complications were uncommon. Renal function generally declined following treatment, but nephron-sparing approaches preserved kidney function better than radical surgery. Early survival outcomes, including recurrence-free and overall survival, were favourable across studies. These findings suggest that neoadjuvant therapy may be a safe and effective strategy to expand surgical options in complex renal tumours. However, larger prospective trials are needed to validate these results.

## Introduction and background

Renal cell carcinoma (RCC) represents approximately 2-3% of all adult malignancies, making it one of the most prevalent urological cancers globally [1-3]. The worldwide incidence of RCC continues to rise, partly due to advances in imaging technologies that have increased the incidental detection of small renal masses [4]. For localised disease, partial nephrectomy (PN) has become the standard of care, as it achieves comparable oncologic outcomes to radical nephrectomy (RN) while preserving renal function and reducing the risk of long-term chronic kidney disease [5-7]. Contemporary international guidelines from the American Urological Association and the European Association of Urology consistently recommend PN for T1 and select T2 tumours whenever technically feasible [8]. Despite these advances, PN remains a technically demanding procedure, particularly for tumours that are anatomically complex, centrally located, or adjacent to vital vascular structures such as the renal hilum [9]. A considerable number of patients present with tumours classified as T2 or T3a, or those with high RENAL or PADUA complexity scores of 10 or greater, which substantially increase operative risk and limit the feasibility of PN [10]. In such cases, surgeons often favour an RN approach to ensure oncologic safety, even though this approach results in a greater loss of functional renal parenchyma and a higher likelihood of postoperative renal insufficiency [11]. This trade-off presents a significant clinical dilemma, especially for patients with solitary kidneys or baseline renal impairment, who face a heightened risk of chronic kidney disease after total nephrectomy. As a result, there is growing clinical interest in preoperative strategies capable of reducing tumour volume and anatomical complexity to increase the feasibility of nephron-sparing surgery in patients with high-complexity or locally advanced RCC.

The concept of neoadjuvant systemic therapy in RCC has emerged as a promising strategy to improve the technical feasibility of PN in patients with anatomically complex or locally advanced disease. The primary goal of such therapy is to achieve tumour downsizing or regression before surgery, thereby facilitating a safer and more complete resection while preserving renal parenchyma. Several classes of systemic agents have been explored for this purpose, most notably vascular endothelial growth factor receptor-targeted tyrosine kinase inhibitors (VEGFR-TKIs) and immune checkpoint inhibitors (IOs). VEGFR-TKIs, such as sunitinib, pazopanib, axitinib, and cabozantinib, inhibit angiogenesis and tumour proliferation by blocking VEGF-mediated signalling pathways, resulting in decreased tumour vascularity and potential volume reduction [12, 13]. On the other hand, immune checkpoint inhibitors (IO), including nivolumab and pembrolizumab, enhance antitumour immune activity by disrupting PD-1/PD-L1 interactions, leading to immune-mediated tumour regression [14].

Early clinical studies have demonstrated that neoadjuvant therapy can reduce tumour size by approximately 10-30%, with several reports showing conversion of patients initially considered ineligible for PN to successful nephron-sparing surgery [15, 16]. Combination approaches integrating VEGFR-TKIs and IO have also demonstrated synergistic effects in metastatic RCC and are now being evaluated in the neoadjuvant setting for locally advanced disease [17]. Despite these encouraging findings, most available evidence is derived from small, single-arm phase II trials and institutional series with heterogeneous patient populations and limited follow-up. Consequently, uncertainty remains regarding the magnitude and consistency of the benefit, the safety profile, and the impact on long-term oncologic outcomes. These limitations highlight the need for comprehensive evidence synthesis to determine whether neoadjuvant systemic therapy truly enhances the feasibility and safety of PN in complex non-metastatic RCC. Although several clinical trials and institutional series have evaluated neoadjuvant systemic therapy for RCC, the available evidence remains limited and fragmented. Most published studies are small, single-arm investigations with heterogeneous patient populations, variable tumour complexity, and inconsistent endpoints, making it difficult to draw firm conclusions about clinical efficacy or safety. Furthermore, outcomes such as PN conversion rate, tumour shrinkage, surgical margins, renal function, and recurrence-free survival are often inconsistently reported. These factors have collectively limited the ability to define the true role of neoadjuvant therapy in complex, non-metastatic RCC [12, 13, 16].

A recent systematic review attempted to synthesise evidence on neoadjuvant therapy but was restricted to VEGFR-targeted TKI, thereby excluding the growing body of literature on IO and emerging combination regimens [18]. To the best of our knowledge, no previous meta-analysis has comprehensively evaluated the collective impact of VEGFR-TKIs, IO monotherapy, and VEGFR-TKI plus IO combinations in this clinical setting. Given the evolving therapeutic landscape and the recent introduction of immunotherapy-based combinations in localised RCC, a broader synthesis is warranted to clarify their comparative safety, surgical feasibility, and oncologic effectiveness. Therefore, this systematic review and meta-analysis aimed to evaluate the effectiveness of neoadjuvant systemic therapy in enabling PN among patients with high-complexity or locally advanced non-metastatic RCC.

## Review

Methodology

Study Design

This systematic review and meta-analysis were conducted in accordance with the guidelines outlined in the Preferred Reporting Items for Systematic Reviews and Meta-Analyses (PRISMA) 2020 statement [19].

Research Question

This review aims to determine whether neoadjuvant systemic therapy (VEGF-TKI, IO, or IO+TKI) increases the feasibility of PN and improves clinical outcomes in adults with high-complexity or locally advanced non-metastatic RCC. The following PICOS format was used and later employed to define the inclusion criteria, aiding in the selection of eligible studies.

Population (P): Adults with non-metastatic RCC (clear cell and non-clear cell) presenting with high-complexity renal masses defined by RENAL, PADUA, and/or hilar involvement; solitary kidney cases included.

Intervention (I): Neoadjuvant systemic therapy administered prior to planned surgical resection, including VEGF-TKIs, IO, or combination IO+TKI regimens.

Comparison (C): Upfront surgery without neoadjuvant therapy, pre-/and post-comparisons within the same neoadjuvant cohort.

Outcome (O): PN rate achieved (conversion/feasibility), surgical margins (R0), tumour size reduction, complications, estimated glomerular filtration rate (eGFR) change, recurrence-free survival, and overall/cancer-specific survival.

Study design (S): Randomised controlled trials (RCTs) and comparative cohort studies; single-arm trials and case series considered for descriptive synthesis of feasibility and outcomes.

Information Sources

A systematic literature search was conducted to identify all relevant studies published in PubMed, Cochrane CENTRAL, Scopus, and ScienceDirect from inception to January 30, 2025. Additional manual searching of reference lists of included articles and relevant reviews was also performed to capture studies not indexed in the primary databases. To ensure comprehensive coverage, both free-text keywords and controlled vocabulary (e.g., MeSH terms in PubMed) were applied. The search combined terms relating to renal cell carcinoma, neoadjuvant systemic therapy (including VEGF-TKIs, IO, and IO+TKI combinations), and PN or kidney-sparing surgery. Boolean operators AND and OR were used to generate the final search strings in each database, and these strategies were adapted to the indexing system of the specific platform. A detailed description of the database-specific search strategies is provided in Table 1.

**Table 1:** Literature search strategies across databases

Database	Search strings	Search results
PubMed	("renal cell carcinoma" OR "kidney cancer" OR RCC) AND (neoadjuvant OR presurgical OR preoperative) AND (axitinib OR sunitinib OR sorafenib OR pazopanib OR "tyrosine kinase inhibitor" OR TKI OR immunotherapy OR "immune checkpoint" OR nivolumab OR pembrolizumab OR atezolizumab OR "VEGF-TKI" OR "VEGFR-TKI") AND (partial nephrectomy OR nephron-sparing OR "partial nephrectom*")	77
Cochrane CENTRAL	("renal cell carcinoma" OR "kidney cancer" OR RCC) AND (neoadjuvant OR presurgical OR preoperative) AND (axitinib OR sunitinib OR sorafenib OR pazopanib OR cabozantinib OR "tyrosine kinase inhibitor*" OR TKI OR immunotherapy OR "immune checkpoint" OR nivolumab OR pembrolizumab OR atezolizumab OR "VEGF-TKI" OR "VEGFR-TKI") AND ("partial nephrectomy" OR "nephron-sparing" OR "nephron sparing" OR "partial nephrectom*")	24
ScienceDirect	("renal cell carcinoma" OR RCC) AND (neoadjuvant OR presurgical) AND (TKI OR immunotherapy) AND ("partial nephrectomy" OR "nephron sparing")	534
Scopus	("renal cell carcinoma" OR "kidney cancer" OR RCC) AND (neoadjuvant OR presurgical OR preoperative) AND (axitinib OR sunitinib OR sorafenib OR pazopanib OR cabozantinib OR "tyrosine kinase inhibitor*" OR TKI OR immunotherapy OR "immune checkpoint" OR nivolumab OR pembrolizumab OR atezolizumab OR "VEGF-TKI" OR "VEGFR-TKI") AND ("partial nephrectomy" OR "nephron-sparing" OR "nephron sparing" OR "partial nephrectom*")	119

Eligibility Criteria

For a study to be considered eligible it had to fulfil the following criteria: (i) written in the English language; (ii) enrolled adults (≥18 years) with non-metastatic RCC; localized (T1-T2) or locally advanced (T3a) tumours; (iii) patients where PN was initially considered challenging or unfeasible due to high anatomical complexity (RENAL or PADUA score ≥10), central/hilar location, solitary kidney, or large tumour size (≥T2); (iv) administered neoadjuvant systemic therapy before surgery, including VEGF-targeted TKI, IO, or combination IO+TKI regimens; (v) reported at least one outcome of interest such as PN feasibility, tumour size reduction, surgical parameters (estimated blood loss, complications), renal function (eGFR), or oncologic endpoints (recurrence-free survival (RFS), overall survival (OS), surgical margins); and (vi) the studies were RCTs, prospective or retrospective cohort studies, or case series with ≥5 patients.

Non-journal articles, non-English publications, case reports (n < 5), conference abstracts, non-full text articles, systematic reviews, meta-analyses, reviews, or other secondary research papers were excluded. Studies focusing exclusively on metastatic RCC, benign tumours, or pediatric populations were also excluded.

Screening of studies was performed independently by three reviewers, who assessed the titles and abstracts of all identified records. Full-text articles were retrieved for studies that appeared to meet the inclusion criteria or where eligibility remained uncertain. Any disagreements were resolved by discussion and consensus; if consensus could not be reached, a fourth reviewer was consulted.

Quality Assessment

The quality of the included studies was assessed using established risk-of-bias tools tailored to the study design. For randomised controlled trials, the Cochrane Risk of Bias 2 (RoB 2) tool was applied, evaluating domains such as the randomisation process, deviations from intended interventions, missing outcome data, measurement of outcomes, and selective reporting [20]. For non-randomised comparative studies, the Risk of Bias in Non-Randomised Studies of Interventions (ROBINS-I) tool was employed, which considers potential bias due to confounding, participant selection, classification of interventions, deviations from intended interventions, missing data, measurement of outcomes, and reporting [21]. Two reviewers independently assessed each study, and discrepancies in ratings were resolved through discussion until a consensus was reached. Where consensus could not be achieved, a third reviewer provided adjudication.

Data Extraction

Data were extracted independently by four reviewers using a standardised Microsoft Excel (Microsoft Corp., Redmond, WA, USA) sheet. Two summary tables were generated to capture study-level and outcome-level information. The study characteristics table included the following items: reference (first author and year), study design, sample size, study setting, proportion of clear cell histology, tumour stage, RENAL or PADUA score, presence of solitary kidney, baseline eGFR (reported as mean ± SD or median with range/IQR), intervention type, specific agents used, treatment duration, and follow-up time. The clinical outcomes table captured key efficacy and safety endpoints, including tumour shrinkage, conversion to PN, operative time, estimated blood loss, surgical margin status (positive or negative), post-treatment eGFR (mean ± SD), recurrence-free or disease-free survival, overall survival, recurrence rate, proportion of patients developing metastasis, and adverse events.

Statistical Analysis

All statistical analyses were performed using Review Manager software (RevMan, version 5.4; Cochrane Collaboration, Oxford, UK) and R software (version 4.4.3; R Foundation for Statistical Computing, Vienna, Austria). For continuous outcomes, the standardised mean difference (SMD) with corresponding 95% confidence intervals (CIs) was calculated as the summary measure. For binary outcomes, results were expressed as risk ratios (RRs) with 95% CIs. For prevalence outcomes, such as pooled PN rate, negative surgical margin rate, tumour shrinkage, and severe complications (Clavien-Dindo ≥III), effect sizes were reported as pooled prevalence estimates with 95% CIs under a random-effects model. All meta-analyses were conducted using the random-effects model to account for expected heterogeneity across studies. Statistical heterogeneity was assessed using the I² statistic, with values of 25%, 50%, and 75% interpreted as low, moderate, and high heterogeneity, respectively. Where high heterogeneity was observed, subgroup analyses were performed to explore potential sources of variability. The level of statistical significance was set at p < 0.05.

Results

Literature Search

**Figure 1:**
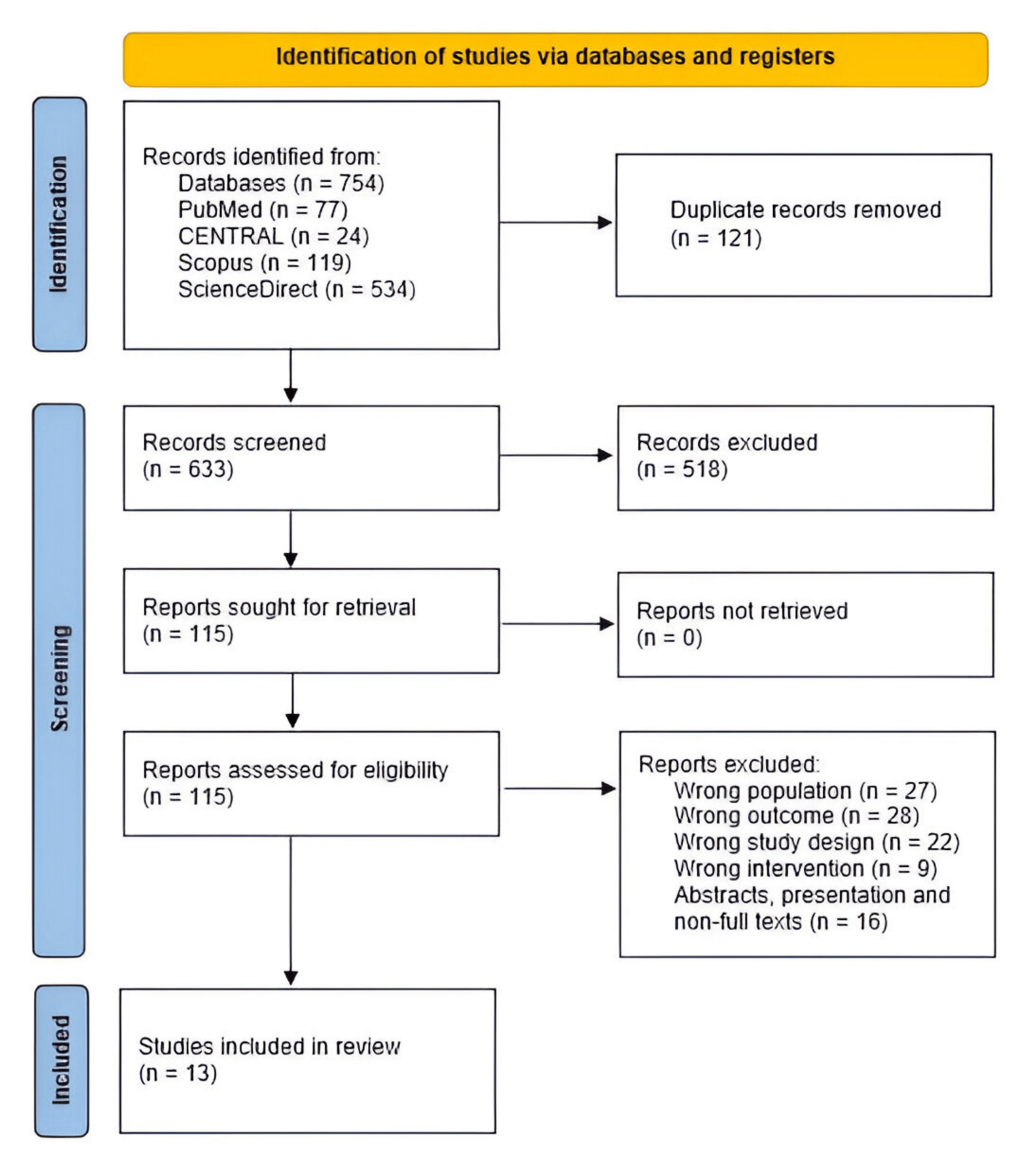
PRISMA flowchart showing study selection process PRISMA: Preferred Reporting Items for Systematic Reviews and Meta-Analyses

The systematic database search yielded a total of 754 records, comprising 77 from PubMed, 24 from Cochrane CENTRAL, 119 from Scopus, and 534 from ScienceDirect. After removing 121 duplicates, 633 unique records remained for screening. Following title and abstract review, 518 studies were excluded as they did not meet the eligibility criteria. The remaining 115 articles underwent full-text assessment, of which 102 were excluded due to reasons such as the presence of metastatic disease, lack of neoadjuvant systemic therapy, or insufficient outcome data. Thirteen studies met the inclusion criteria and were incorporated into this systematic review and meta-analysis. This procedure is shown in Figure 1 below.

Baseline Characteristics

This systematic review and meta-analysis included 13 studies: two randomised controlled trials and 11 non-randomised trials, with a total study population of 359 patients. The majority of included patients had clear cell RCC, with several studies reporting nearly 100% clear cell histology. Tumour stages ranged from localised (T1-T2) to locally advanced (T3/T4) disease, with some studies also including patients with high anatomical complexity defined by RENAL or PADUA scores ≥10. Regarding treatment regimens, 10 studies investigated VEGFR-targeted TKI monotherapy (such as sunitinib, pazopanib, axitinib, sorafenib, or cabozantinib). Two studies evaluated IO monotherapy (nivolumab), while one study assessed a combination of IO (nivolumab) and TKI (axitinib plus toripalimab). Treatment durations ranged from 2 to 16 weeks, and median follow-up times ranged from 1 month to over 25 months. A detailed summary of the study and patient characteristics is provided in Table 2.

**Table 2:** Baseline characteristics of included studies VEGF-TKI: vascular endothelial growth factor tyrosine kinase inhibitor; IO: immune checkpoint inhibitor; w: week; RCT: randomised controlled trial; RCC: renal cell carcinoma; eGFR: estimated glomerular filtration rate; NR: not reported

Reference	Study design	Sample size	Setting	Clear cell histology	Tumour stage	RENAL/PADUA Score	Solitary kidney	Baseline eGFR (mean ± SD or median)	Intervention type	Agents used	Treatment duration	Follow-up time
Bilen et al., 2025 [22]	Phase 2, open-label	17	United States	100%	Clinical stage T3/T4	NR	NR	NR	VEGFR-TKI	Cabozantinib 60 mg daily	12 w	Median follow-up: 25 Months
Huang et al., 2024 [23]	Phase 2, open-label	20	China	100%	T2- T3N0- 1M0	NR	NR	NR	VEGFR-TKI + IO	Axitinib 5 mg twice daily, Toripalimab 240 mg on day 1 of a 3-week, 6-week, and 9-week cycle	12 w	Median follow-up: 21.3 Months
Hakimi et al., 2024 [24]	Phase 2, open-label	27	United States	100%	Clinical stage T2–T3M0, N0-1	RENAL score 10-12 (high complexity)	6 patients (22.2%) had a solitary kidney	81.4% of patients had baseline eGFR <60 mL/min/1.73 m²	VEGFR-TKI	Axitinib 5 mg twice daily	8 w	30 days
Carlo et al., 2022 [25]	Prospective	18	United States	100%	cT1–cT3 localized	NR	NR	NR	IO	Nivolumab	2 w	22.7 months
Gorin et al., 2022 [26]	Prospective	17	United States	94%	T2a–T4N (any) M0 or T (any) N1M0	NR	NR	NR	IO	Nivolumab 3 mg/kg	2 w	24.7 months
Voylenko et al., 2021 [27]	RCT	58	Ukraine	100%	T1-T2 N0 M0	NR	NR	NR	VEGFR-TKI	Pazopanib 800 mg daily orally or sunitinib 50 mg daily for two cycles	4 w	24 months
Okamura et al., 2019 [28]	Retrospective	9	Japan	83%	Advanced RCC with level III–IV IVC thrombus	NR	NR	NR	VEGFR-TKI	Pazopanib 800 mg oral	12 w	30 days
Lebacle et al., 2018 [15]	Phase 2, open-label	18	France	100%	T2-T3N0M0	RENAL score: 10–11	NR	Mean eGFR: 99.8 mL/min/1.73 m²	VEGFR-TKI	Axitinib 5 mg twice daily	2–6 months	24 months
McDonald et al., 2017 [16]	Retrospective	47	United States	100%	Complex renal masses (RENAL scores 10–11), T stage ≥cT2	RENAL score: 10–11	15 patients (31.9%) had a solitary kidney	Median preoperative eGFR 67.1 mL/min/1.73 m2	VEGFR-TKI	Sunitinib 50 mg or 37.5 mg daily	4 -6 w	25 months
Hatiboglu et al., 2017 [29]	RCT	9	Germany	83%	T1–3N0M0	RENAL score 8.5	NR	NR	VEGFR-TKI	Sorafenib 400 mg	4 w	24 months
Rini et al., 2015 [12]	Phase 2, open-label	25	United States	96%	cT1–cT2, N0, M0	RENAL score: 10–12	14 patients (56%) had a solitary kidney	Median eGFR: 64 mL/min/1.73 m²	VEGFR-TKI	Pazopanib 800 mg daily	16 w	NR
Karam et al., 2014 [13]	Phase 2, open-label	22	United States	100%	cT3aN0M0	NR	NR	NR	VEGFR-TKI	Axitinib 5mg twice a day	12 w	NR
Lane et al., 2014 [30]	Retrospective	72	United States	90%	cT1a, cT1b, cT2a, cT2b, cT3/T4	RENAL score: 10	18 patients (25%) had a solitary kidney	NR	VEGFR-TKI	50-mg sunitinib	6 w	22 months

Quality Assessment

**Figure 2:**
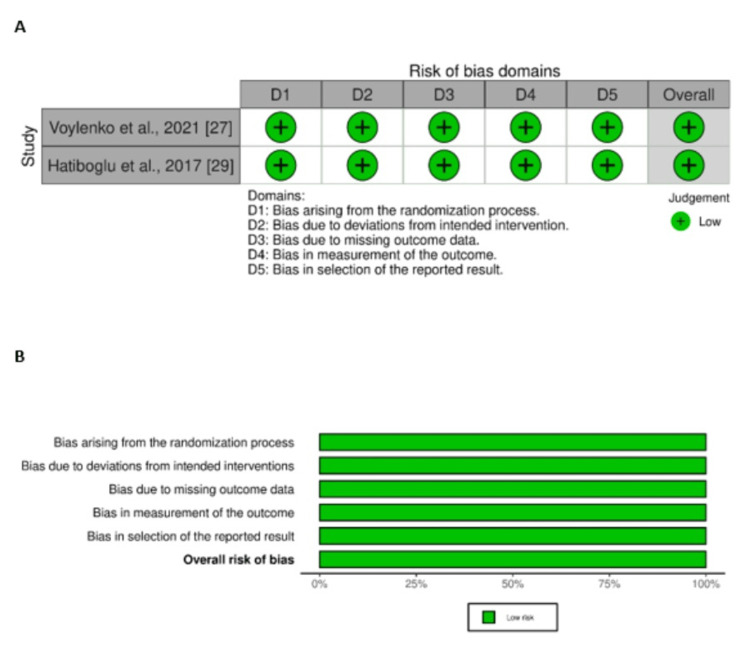
Risk of bias assessment for randomised controlled trials (A) Traffic plot and (B) summary plot

**Figure 3:**
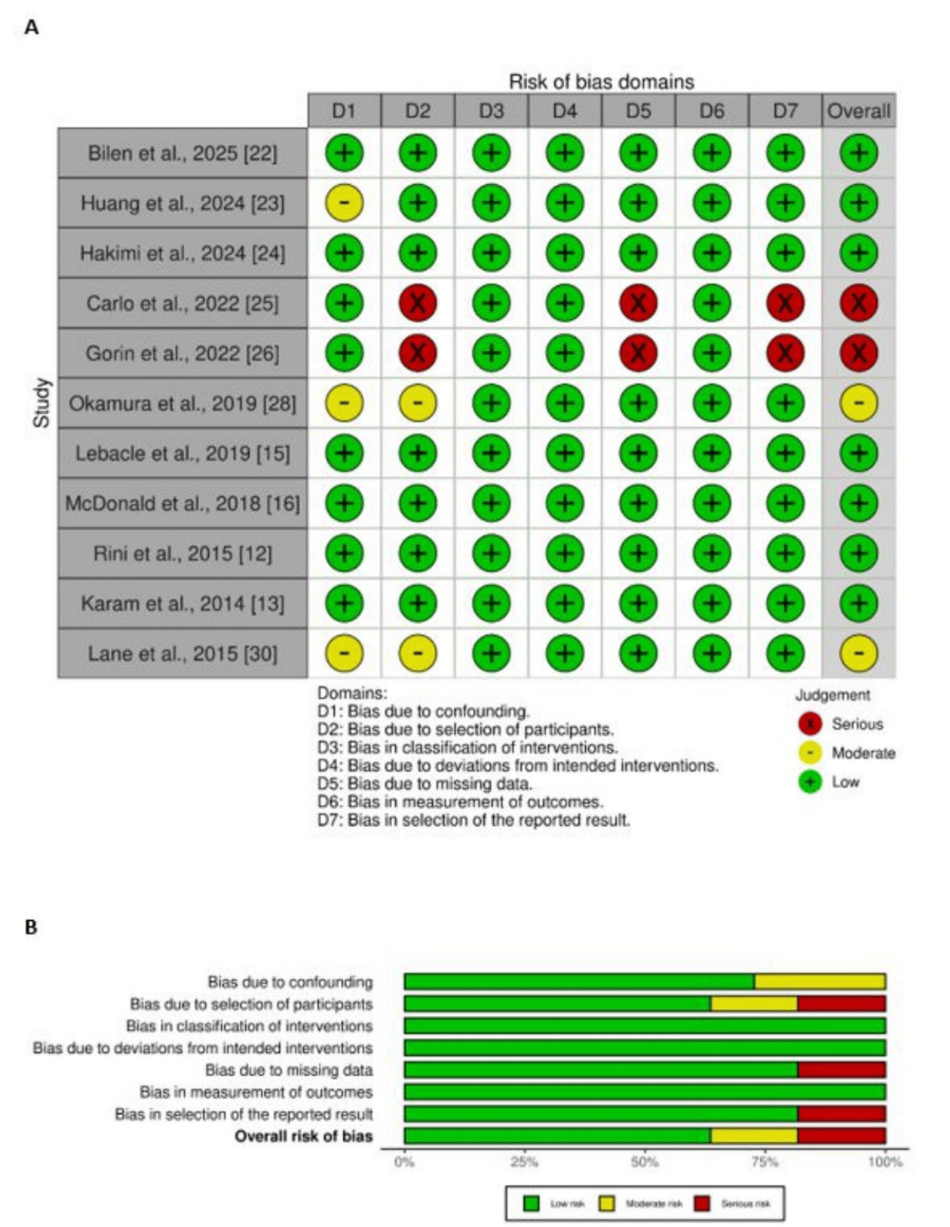
Risk of bias assessment for non-randomised studies (A) Traffic plot and (B) summary plot

Risk of bias was evaluated separately for randomised and non-randomised studies. The two RCTs demonstrated an overall low risk of bias, as shown in the RoB 2 traffic and summary plots. Among the eleven non-randomised studies assessed with ROBINS-I, seven were judged to have a low risk of bias, two a moderate risk, and two a serious risk of bias. The corresponding traffic and summary plots for both RCTs and non-randomised studies are presented in Figure 2 and Figure 3.

Clinical Outcomes

Across the 13 included studies, clinical outcomes varied but consistently addressed feasibility and surgical safety. Reported endpoints included tumour shrinkage following neoadjuvant therapy, conversion to PN, operative time, blood loss, surgical margin status, renal function preservation, recurrence, OS, and adverse events. A detailed summary of these findings is presented in Table 3.

**Table 3:** Clinical outcomes reported across included studies OS: overall survival; DFS: disease-free survival; RFS: recurrence free survival; PN: partial nephrectomy; eGFR: estimated glomerular filtration rate; Aes: adverse events; min: minutes; cc: cubic centimetre; cm: centimetre; AKI: acute kidney injury; MFS: metastasis-free survival; NR: not reported; IQR: interquartile range

Study	Sample size	Tumour shrinkage	Conversion to PN	Operative time	Blood loss	Surgical margins (+/-)	Post-treatment eGFR (mean ± SD)	RFS/DFS	OS	Recurrence	Metastasis (%)	Adverse events
Bilen et al., 2025 [22]	17	Median tumour reduction 26% (range 8–42%)	2 (12.5%)	NR	NR	Negative	NR	Median follow-up 25 months; 1-year DFS 82.4% (95% CI 54.7–93.9%)	1-year OS 94.1% (95% CI 65–99.1%)	1 patient deceased from RCC	0	Most common AEs: Diarrhoea, nausea, fatigue, hypertension, anorexia, palmar-plantar erythrodysesthesia; no grade 4–5 AEs
Huang et al., 2024 [23]	20	Median 26.7% tumour reduction (range -2.0% to 40.3%)	1 (5.3%)	160 min (range: 80–420)	300 mL (range: 40–2500)	Negative	NR	1-year DFS: 84.7% (95% CI: 68.7%-100%)	NR	4 (20%)	Lung and liver metastasis in 1 patient	Most common AEs: Hypertension (35%), Dysphonia (30%), Stomatitis (25%), Fatigue (15%), Hand-foot syndrome (15%)
Hakimi et al., 2024 [24]	27	Median 19% tumour reduction	20 (74%)	NR	NR	25/27 patients had negative margins	3 patients (11.1%) had ≥50% reduction in eGFR	NR	NR	1 (3.7%)	NR	Grade 3 AEs: Hypertension (11.1%), fatigue (3.7%), diarrhoea (0%), proteinuria (3.7%)
Carlo et al., 2022 [25]	18	NR	NR	Median operative time 174 min (range 158–195)	Median estimated blood loss 200 mL (150–363)	Negative	NR	RFS 1 year: 82% (95% CI 65–100%)	NR	NR	NR	2 patients discontinued nivolumab early due to immune-related adverse events (irAEs): grade 3 transaminitis and grade 2 arthralgia; one late grade 3 colitis/AKI attributed to nivolumab after 6 months
Gorin et al., 2022 [26]	17	NR	NR	NR	NR	NR	NR	NR	1-year MFS 100%, OS 100%; 2-year MFS 85.1%, OS 100%; 3-year MFS 85.1%, OS 85.7%	NR	2 (11.76%)	14/17 patients (82.4%) had 49 AEs; 2 patients (11.8%) had grade 3 AEs; no grade 4–5 AEs. Common AEs (grade 1) included fatigue 41.2%, pruritus 29.4%, rash 29.4%
Voylenko et al., 2021 [27]	58	Median tumour regression 20.5% ± 14.3% (range 0–60%)	91.4% PN in the TT group; 33.3% PN in the surgery alone group	NR	NR	Negative	NR	NR	NR	NR	NR	NR
Okamura et al., 2019 [28]	9	NR	NR	497 min	1928 mL	NR	NR	NR	NR	NR	NR	Thrombocytopenia: 2 patients (22%) Hand-foot syndrome: 1 patient (11%)
Lebacle et al., 2019 [15]	18	Median 17.1% tumour reduction	12 (67%)	Median 227 min (range: 110-365)	Median 600 mL (range: 70-2400)	16/18 patients had negative margins	Median eGFR: 87 mL/min/1.73 m²	NR	NR	1 (5.56%)	1 (5.56%)	12 with grade 1/2 and five with grade 3. The most frequent AEs were hypertension, fatigue, dysphonia, and hand–foot syndrome. There were no grade 4 or 5 AEs
McDonald et al., 2018 [16]	47	Median tumour size pre-post: 14 cm	NR	NR	NR	45/47 patients had negative margins	Median eGFR: 60.7 mL/min/1.73 m²	NR	NR	NR	NR	14/47 patients (29.8%) experienced high-grade toxicity during neoadjuvant sunitinib
Hatiboglu et al., 2017 [29]	9	Median reduction 29% (range: -4.9-61.1)	4 (44.4%)	NR	NR	2/9 patients had negative margins	NR	NR	NR	NR	NR	Hand-foot syndrome (4/9), hypertension (1/9), and generalised exanthema (1/9); total Grade 3 events = 6 across 9 patients; dose reductions were applied.
Rini et al., 2015 [12]	25	Median 7.3 cm	6 (46.2%)	NR	NR	23/24 patients had negative margins	8.9 mL/min/1.73 m²	NR	NR	NR	NR	16 patients (64%) reported grade 3 AEs, primarily hypertension in 9 (36%) and elevated liver enzymes in 5 (20%)
Karam et al., 2014 [13]	22	Median reduction 28.3% (range: 5.3-42.9)	5 (22.7%)	87 min	225 cc	NR	NR	NR	NR	NR	NR	No grade 4 or grade 5 AEs were observed. The most common grade 3 AE was hypertension.
Lane et al., 2015 [30]	72	Median tumour size reduced from 7.2 cm → 5.3 cm Median reduction in diameter 18% (IQR 7–27%)	49 kidneys (63%)	NR	NR	NR	NR	NR	2-year OS 89.3% 5.1%	7 (9.7%)	3 (4.17%)	Grade ≥ 3 surgical complications occurred in 5 patients (7%)

Oncological Outcomes

**Figure 4:**
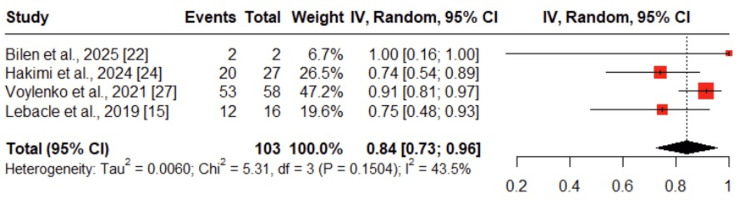
Forest plot of pooled partial nephrectomy (PN) rate after neoadjuvant therapy The figure presents the pooled PN rate derived from studies [15], [22], [24], and [27], showing the proportion of patients who successfully underwent PN following neoadjuvant therapy

PN rate achieved: This outcome was reported in four studies, including a total of 103 patients [15, 22, 24, 27]. The pooled analysis showed that neoadjuvant therapy enabled successful PN in 84% of cases (95% CI: 0.73-0.96), with moderate heterogeneity (I² = 43.5%) (Figure 4).

Surgical Margins

**Figure 5:**
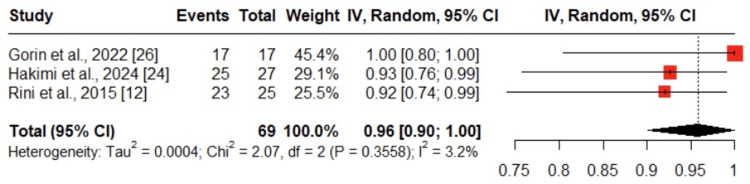
Forest plot of pooled negative surgical margin (R0) rate after neoadjuvant therapy The figure illustrates the pooled rate of achieving negative surgical margins (R0 resection) following neoadjuvant therapy, based on data from studies [12], [24], and [26]

This outcome was evaluated in three studies, including a total of 69 patients who underwent PN after neoadjuvant therapy [12, 24, 26]. The pooled analysis demonstrated that 96% achieved negative surgical margins (95% CI: 0.90-1.00), indicating a high rate of oncologic adequacy. Statistical heterogeneity was low (I² = 3.2%) (Figure 5).

RFS and overall/cancer-specific survival: Data on RFS and survival outcomes were reported in a limited number of studies. Bilen et al. reported, at a median follow-up of 25 months in 17 treated patients, a one-year RFS of 82.4% (95% CI: 54.7-93.9%) and a one-year OS of 94.1% (95% CI: 65.0-99.1%) [22]. In another prospective study, Carlo et al. observed a one-year RFS of 82% (95% CI: 65-100%) [25]. Gorin et al. reported particularly favourable outcomes, with metastasis-free survival and OS both at 100% at 1 year, 85.1% and 100% at 2 years, and 85.1% and 85.7% at 3 years, respectively, at a median follow-up of 24.7 months [26]. In the cohort studied by Huang et al., with a median follow-up of 21.3 months (range 5.9-35.1 months) and no adjuvant therapy administered, four patients experienced recurrence. However, the median disease-free survival (DFS) was not reached. The estimated DFS at 1 and 2 years was 84.7% (95% CI: 68.7-100%) [24].

Surgical Outcomes

**Figure 6:**
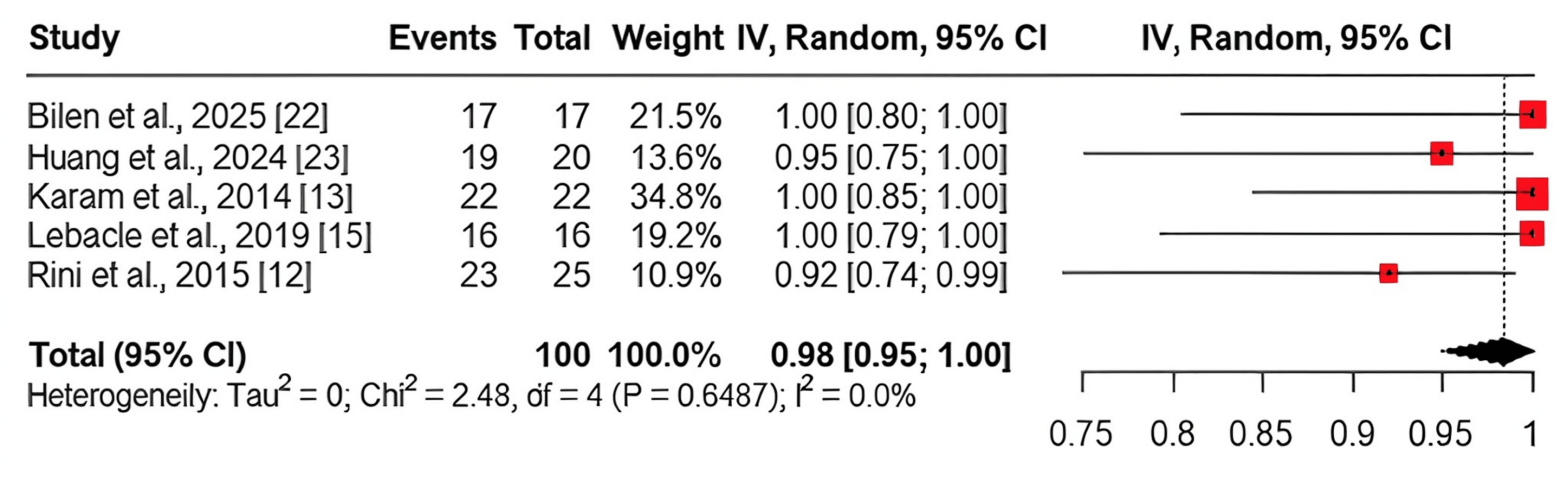
Forest plot of pooled tumour shrinkage (tumour size reduction) after neoadjuvant therapy This figure displays the pooled estimate of tumour size reduction following neoadjuvant therapy, based on data from studies [12], [13], [15], [22], and [23]

Tumour size reduction: This outcome was assessed in five studies [12, 13, 15, 22, 23]. The pooled analysis revealed a high prevalence of tumour shrinkage, with a rate of 0.98 (95% CI: 0.95-1.00). Statistical heterogeneity was negligible (I² = 0%) (Figure 6).

**Figure 7:**
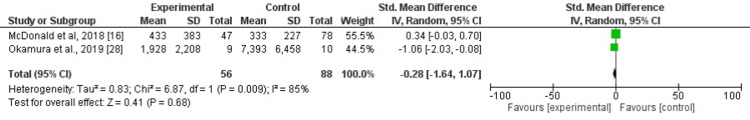
: Forest plot comparing estimated blood loss (mL) between neoadjuvant therapy and upfront surgery The figure compares mean estimated blood loss between patients who received neoadjuvant therapy and those who underwent upfront surgery, using data from studies [16] and [28]

Blood loss: Two studies compared estimated blood loss between patients treated with neoadjuvant therapy and those undergoing upfront surgery [16, 28]. The pooled analysis yielded an SMD of -0.28 (95% CI: -1.64 to 1.07); p = 0.68, indicating no statistically significant difference between the two groups. However, heterogeneity was high (I² = 85%) (Figure 7).

**Figure 8:**
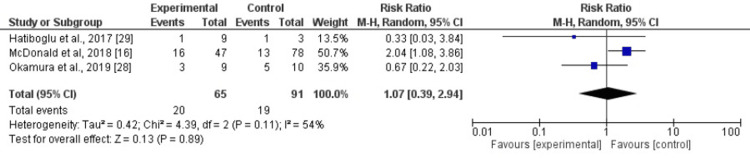
: Forest plot comparing perioperative complications between neoadjuvant therapy and upfront surgery This figure presents the pooled comparison of perioperative complication rates between patients undergoing neoadjuvant therapy and those receiving upfront surgery, based on data from studies [16], [28], and [29]

Perioperative complications: Three studies compared perioperative complications between patients receiving neoadjuvant therapy and those undergoing upfront surgery [16, 28, 29]. The pooled analysis demonstrated a RR: 1.07 (95% CI: 0.39-2.94; p = 0.89) (Figure 8). Moderate heterogeneity was observed (I² = 54%).

**Figure 9:**
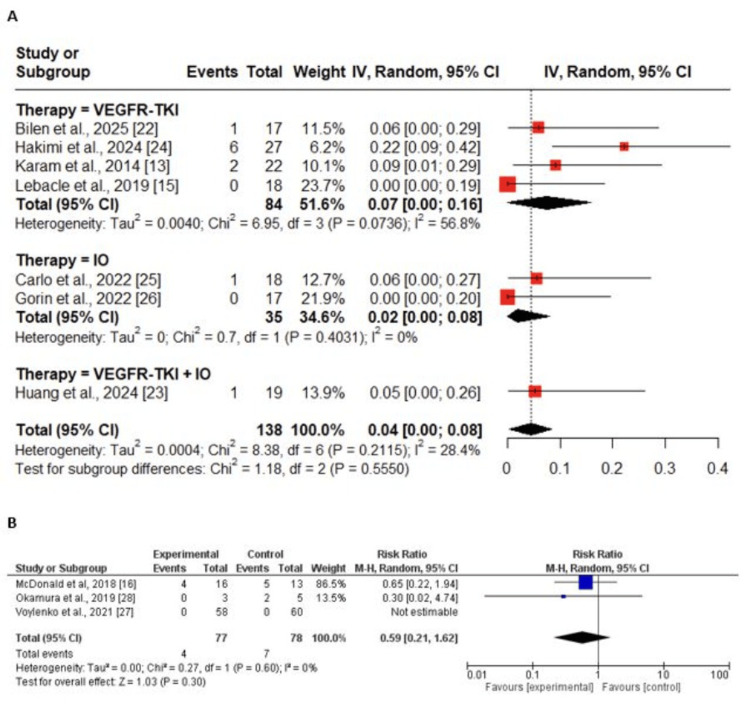
Forest plots of Clavien–Dindo grade ≥III complications after neoadjuvant therapy Pooled prevalence of Clavien–Dindo ≥III complications within neoadjuvant therapy cohorts, with subgroup analyses by therapy type. (B) Comparison of Clavien–Dindo ≥III complications between neoadjuvant therapy and upfront surgery. Data are derived from studies [13], [15], [22-26] for panel A and [16], [27], [28] for panel B

Complications with a grade of ≥3 (Clavien‑Dindo): The analysis of severe complications (grade ≥ III) showed a low overall prevalence in neoadjuvant therapy cohorts, with a pooled rate of 0.04 (95% CI: 0.00-0.08; I² = 28.4%) across seven studies (Figure 9A). Subgroup analysis indicated slightly higher rates with VEGFR-TKI therapy (0.07; 95% CI: 0.00-0.16) compared to IO monotherapy (0.02; 95% CI: 0.00-0.08), while the single study of VEGFR-TKI + IO combination reported a prevalence of 0.05 (95% CI: 0.00-0.26). In comparative studies, there was no significant difference between neoadjuvant therapy and upfront surgery, with a pooled RR of 0.59 (95% CI: 0.21-1.62; p = 0.30; I² = 0%) (Figure 9B).

Renal Outcomes

eGFR change: Renal functional outcomes were variably reported across the included studies, with most demonstrating a decline in eGFR following neoadjuvant therapy and subsequent surgery, although the magnitude of this decline differed. Hakimi et al. found small but significant changes at last follow-up, with median serum creatinine increasing from 1.25 to 1.48 mg/dL (p = 0.005) and median eGFR decreasing from 48.5 to 40.0 mL/min/1.73 m² (p < 0.001), corresponding to a median decline of 8.5 mL/min/1.73 m² [24]. In the series by Lane et al., the baseline GFR for the entire cohort was 63 mL/min/1.73 m², declining to 51 mL/min/1.73 m² at a median follow-up of 2.2 years. Stratified by treatment, GFR change was +5% in the non-surgical group, -16% after PN, and -24% after RN (p = 0.029), confirming better preservation with PN [30]. Lebacle et al. reported a median eGFR of 87 mL/min one month after surgery, representing an early decline of 11 mL/min compared to baseline (p = 0.011), and a mean decrease of 13 mL/min at 24 months (p = 0.001) [15]. Similarly, Rini et al. observed a median decrease in eGFR of 22.2% overall and 16.2% in patients undergoing PN, corresponding to absolute declines of 8.9 mL/min/1.73 m² and 6.2 mL/min/1.73 m², respectively [12].

Discussion

Neoadjuvant therapy in patients with high-complexity RCC has demonstrated promising potential in enabling PN, as evidenced by our pooled analysis. This study showed that neoadjuvant systemic therapy, including VEGFR-TKIs, IO, and their combinations, significantly increased the feasibility of PN in previously inoperable or high-risk cases. The pooled prevalence of PN achieved across studies was notably high, with a substantial reduction in tumour size and the ability to achieve negative surgical margins. These findings suggest that neoadjuvant therapy can effectively downsize tumours, improve resectability, and enhance the likelihood of nephron-sparing surgery in patients who would otherwise require RN.

Findings from this systematic review and meta-analysis suggest that neoadjuvant systemic therapy is an effective strategy for increasing the feasibility of PN in patients with high-complexity RCC. The pooled analysis revealed high rates of tumour shrinkage and negative surgical margins, which are critical indicators of oncologic success and surgical adequacy. These results support the hypothesis that neoadjuvant therapy, particularly VEGFR-TKIs, contributes to the downstaging of tumours, making them amenable to nephron-sparing procedures [15, 16, 28]. However, moderate heterogeneity across studies, especially in the response to IO, suggests that while the overall trend is positive, variability in outcomes exists due to differing patient characteristics and treatment regimens. Despite the positive outcomes, IO showed less consistent results compared to VEGFR-TKIs [25, 26]. This variability can likely be attributed to the immune-related mechanisms of action, which can differ among patients and tumour types. In some studies, the combination of immune checkpoint inhibitors and VEGFR-TKI therapy showed promising results, but further investigation is needed to define the optimal therapeutic regimens [23]. Additionally, the relatively low rate of adverse events observed in the studies is encouraging. However, the high heterogeneity seen in some analyses underscores the need for more controlled trials to refine treatment protocols and evaluate safety in a broader range of patient populations. Overall, neoadjuvant therapy appears to be a viable option for enhancing surgical outcomes; however, careful patient selection and individualised treatment strategies will be crucial to maximise its benefits.

Our study corroborates and extends the current understanding of neoadjuvant therapy in RCC. Previous studies have predominantly focused on single-agent therapies, particularly VEGFR-TKIs. For instance, a systematic review by Zhu et al. demonstrated that neoadjuvant VEGFR-TKI treatment significantly increased the proportion of patients undergoing partial nephrectomy, reduced operative time, and decreased blood loss, without increasing perioperative complications [18, 31, 32]. These findings are consistent with our pooled analysis, which also reported high rates of partial nephrectomy and favourable surgical outcomes following neoadjuvant therapy. However, our study extends the current understanding by incorporating IOs and combination therapies (VEGFR-TKI plus IO) into the analysis. Notably, a recent review by Qin et al. highlighted the evolving role of IOs and combination regimens in the neoadjuvant setting for RCC, emphasising the need for further investigation into their efficacy and safety [33]. Our inclusion of these therapies provides a more comprehensive assessment of neoadjuvant strategies and their impact on surgical feasibility and oncologic outcomes. While our findings support the use of neoadjuvant therapy to facilitate partial nephrectomy in high-complexity RCC, they also underscore the variability in treatment responses, particularly with IO monotherapy. This variability aligns with observations from other studies, such as Marandino et al., who noted that IO monotherapy has shown less consistent results in the neoadjuvant setting compared to VEGFR-TKI monotherapy [34]. These differences highlight the necessity for personalised treatment approaches and further research to identify predictive biomarkers that can guide therapy selection.

Neoadjuvant systemic therapy has demonstrated substantial potential in increasing the feasibility of PN, even in patients initially considered unsuitable for nephron-sparing surgery. This finding supports the adoption of neoadjuvant therapy as a strategy to expand the surgical options available to patients with complex renal tumours, thereby avoiding the need for RN and preserving renal function in at-risk patients, such as those with a solitary kidney or pre-existing renal impairment. The high rates of tumour shrinkage, negative surgical margins, and partial nephrectomy conversion observed in our analysis are promising for the future of kidney-sparing surgery in high-risk RCC patients. These outcomes are particularly relevant for improving long-term renal function, as nephron-sparing surgery has been shown to have better renal preservation compared to RN. The relatively low incidence of severe complications (Clavien-Dindo grade ≥III) suggests that neoadjuvant therapy is a safe approach, with no significant increase in surgical morbidity compared to upfront surgery. This finding underscores the potential role of systemic therapy in improving not only oncologic outcomes but also perioperative safety. While IO showed variable results compared to VEGFR-TKIs, their inclusion in combination regimens holds promise for further improving treatment outcomes. The ongoing development of combination therapies could potentially enhance the efficacy of neoadjuvant approaches, providing a multimodal strategy that targets both tumour biology and the immune environment, thus expanding the pool of patients who are candidates for PN. However, as observed in our analysis, careful patient selection remains crucial, particularly in deciding between single-agent IO or combination therapy, depending on individual tumour biology and response.

Study Limitations

One of the strengths of this study is that it is the first comprehensive meta-analysis to include VEGFR-TKIs, IO, and IO+TKI combinations, providing a broader understanding of neoadjuvant therapy in high-complexity RCC. Additionally, our study employed a robust methodological approach, synthesising data from both randomised and non-randomised studies to offer a more comprehensive assessment of treatment efficacy and safety. However, several limitations should be acknowledged. The majority of studies included were small, single-arm trials, which may introduce bias and limit the generalizability of the findings. High heterogeneity was observed in some outcomes, reflecting variability in treatment protocols and patient populations. Additionally, the inclusion of non-randomised studies could lead to potential confounding factors, which may affect the strength of the conclusions drawn.

## Conclusions

The effectiveness of neoadjuvant systemic therapy with VEGFR-TKIs, immune checkpoint inhibitors, and combination regimens has shown promising results in increasing the feasibility of PN in patients with high-complexity or locally advanced RCC. Neoadjuvant therapy has the potential to achieve substantial tumour shrinkage, increase negative surgical margins, and improve oncologic and renal functional outcomes compared to RN. Despite the promising findings, further RCTs and large-scale studies are necessary to optimise treatment regimens and definitively confirm the long-term benefits of neoadjuvant therapy in this patient population.
